# Optimization of SOX2 Expression for Enhanced Glioblastoma Stem Cell Virotherapy

**DOI:** 10.3390/sym16091186

**Published:** 2024-09-10

**Authors:** Dongwook Kim, Abraham Puig, Faranak Rabiei, Erial J. Hawkins, Talia F. Hernandez, Chang K. Sung

**Affiliations:** 1 Department of Mathematics, College of Arts and Sciences, Texas A&M University-Kingsville, Kingsville, TX 78363, USA; 2 Department of Biological and Health Sciences, College of Arts and Sciences, Texas A&M University-Kingsville, Kingsville, TX 78363, USA

**Keywords:** glioblastoma, cancer stem cells, virotherapy, Zika virus, SOX2, mathematical model, dynamical system

## Abstract

The Zika virus has been shown to infect glioblastoma stem cells via the membrane receptor $\alpha_{v}\beta_{5}$, which is activated by the stem-specific transcription factor SOX2. Since the expression level of SOX2 is an important predictive marker for successful virotherapy, it is important to understand the fundamental mechanisms of the role of SOX2 in the dynamics of cancer stem cells and Zika viruses. In this paper, we develop a mathematical ODE model to investigate the effects of SOX2 expression levels on Zika virotherapy against glioblastoma stem cells. Our study aimed to identify the conditions under which SOX2 expression level, viral infection, and replication can reduce or eradicate the glioblastoma stem cells. Analytic work on the existence and stability conditions of equilibrium points with respect to the basic reproduction number are provided. Numerical results were in good agreement with analytic solutions. Our results show that critical threshold levels of both SOX2 and viral replication, which change the stability of equilibrium points through population dynamics such as transcritical and Hopf bifurcations, were observed. These critical thresholds provide the optimal conditions for SOX2 expression levels and viral bursting sizes to enhance therapeutic efficacy of Zika virotherapy against glioblastoma stem cells. This study provides critical insights into optimizing Zika virus-based treatment for glioblastoma by highlighting the essential role of SOX2 in viral infection and replication.

## 1. Introduction

Glioblastoma multiforme (GBM), commonly known as glioblastoma, is one of the most frequent and malignant of all central nervous system (CNS) tumors [1–3]. This aggressive cancer currently leads to a life expectancy of 15 months once diagnosed, with fewer than 5% of patients surviving for five years after diagnosis [4–6]. Glioblastoma prognosis is extremely poor due to its resistance to treatment and its aggressive nature [7,8]. Patients can experience a diverse range of symptoms, such as increased intracranial pressure, headaches, focal or progressive neurologic deficits, and seizures [2]. The treatments used for malignant primary brain tumors are radiotherapy with concomitant chemotherapy using the alkylating agent temozolomide and surgical resection [2,8,9]. Despite available treatments to inhibit tumor growth, GBM remains incurable [3]. According to the World Health Organization (WHO), GBM is classified as a grade IV glioma, the highest grade [6,10,11].

Cancer stem cells are known for their pluripotent and self-renewing properties, contributing to the initiation and progression of tumors [7]. Glioblastoma stem cells (GSCs) play an important role in mediating therapeutic resistance and recurrence in glioblastoma [7,9]. If not eliminated during chemotherapy, GSCs tend to reinitiate tumor formation, causing tumor recurrence [12]. Therefore, the development of novel therapeutic strategies to specifically target GSC populations for elimination could have significant clinical impacts [7].

In oncolytic virotherapy (OVT), lytic viruses are engineered to specifically target cancer cells, leading to viral replication within cancer cells and host-cell lysis [13,14]. In contrast to conventional radiation therapy or chemotherapy, OVT minimizes damage to normal cells and tissues, offering a more targeted and tolerable therapeutic approach [13–15]. The first FDA-approved virotherapy, Talimogene laherparepvec (Amgen Inc., Thousand Oaks, CA, USA), utilizes an engineered herpes simplex virus to target and lyse melanoma cancer cells [16]. Various types of oncolytic viruses have been employed to treat GBM. Among these, the mutated herpes virus G47 has been clinically used for recurrent glioblastoma, with its usage approved in Japan [17]. Recently, Ling et al. reported their phase I trial data involving 41 GBM patients, demonstrating that their engineered herpes virus CAN-3110 could induce T cells and improve patient survival [18].

Zika virus (ZIKV) belongs to the flavivirus genus of RNA viruses and infects the central nervous system by targeting neural precursor cells, ultimately leading to cellular death [19]. ZIKV is known to cause neurodevelopmental disruptions and brain abnormality in fetuses, including microcephaly [19]. While ZIKV can replicate in adult brain tissue and target mature neurons [20], the symptoms following viral infection in adults generally appear to be minimal [21]. Due to the differentiation, proliferation, and cell death observed during ZIKV infection, coupled with the smaller number of negative effects seen in adults, researchers have explored the potential use of ZIKV as a virotherapeutic agent in various neural cancers, including glioma [22,23], neuroblastoma [24,25], and glioblastoma [26–29].

Several mathematical models have been developed to elucidate the intricate interactions between cancer cells and oncolytic viruses employing ordinary differential equations (ODEs) [30–33] and partial differential equations (PDEs) [19,34,35], incorporating time delays to address the lytic cycle of viruses [34–36]. Furthermore, studies have proposed the anti-viral and anti-tumoral effects of immune responses on OVT [37–40]. Our previous study was to investigate the role of natural killer (NK) cells on OVT where equilibrium points can be created or destroyed by activation of NK cells [37]. Recent experimental studies [27–29]show that ZIKV selectively infects GSCs via the membrane receptor $\alpha_{v}\beta_{5}$, which is highly expressed by the transcription factor SOX2 in GSCs. This positive correlation between the levels of SOX2 expression and ZIKV infection suggests the potential utility of SOX2 as a therapeutic marker for predicting successful treatment outcomes. This also underscores the significance of monitoring SOX2 levels during ZIKV virotherapy to optimize therapeutic approaches. Although the complex interplays between cancer cells and oncolytic viruses have been investigated, no mathematical model has been developed to address the dynamic interaction between cancer stem cells and Zika viruses.

In this study, we developed a mathematical ODE model to understand the underlying mechanism of interaction among GSCs, infected GSCs, and ZIKV. We focused on examining the effect of SOX2 expression levels and various viral bursting sizes on the outcome of OVT to identify the optimal parameters for maximizing the efficacy of ZIKV–GSC virotherapy. Our numerical results showed that (1) the efficacy of the therapy was high as the SOX2 expression level was high, (2) two bifurcation values for both SOX2 expression level and viral bursting size were observed in GSC population dynamics, and (3) viral bursting size had symmetry related to the SOX2 expression level in terms of the stability of equilibrium points.

## 2. Materials and Methods

### 2.1. Model

Experimental studies have shown that ZIKV-infected GSCs express the stem cell maker SOX2, with more than 90% of infected cells being SOX2^+^ [28]. The level of SOX2 expression correlates with susceptibility to ZIKV infection, highlighting SOX2 as a key determinant in the interaction between ZIKV and GSCs [27,29]. In this model, we assume that (1) GSCs proliferate with the logistic growth rate $\left(\lambda \right)$, up to their carrying capacity [27,41], (2) oncolytic ZIKV is 100% GSC-specific, (3) one virus particle infects one GSC; if a virus enters a GSC, it is incapable of infecting additional GSCs and ceases to be part of the free virus population, and (4) SOX2 expression level is included in the rate of cells infected by ZIKV. The three-dimensional model of GSCs with ZIKV is given by

(1) $$\frac{dx\left(t\right)}{dt}=\lambda x\left(t\right)\left(1-\frac{x\left(t\right)}{K}\right)-asx\left(t\right)v\left(t\right)\;\frac{dy\left(t\right)}{dt}=asx\left(t\right)v\left(t\right)-\delta_{1}y\;\frac{dv\left(t\right)}{dt}=b\delta_{1}y\left(t\right)-asx\left(t\right)v\left(t\right)-\delta_{2}v\left(t\right)$$

- $x\left(t\right)$ is the population of GSCs;
- $y\left(t\right)$ is the population of GSCs infected by ZIKV and the subpopulation of $x\left(t\right)$;
- $v\left(t\right)$ is the free Zika viruses;
- The term $\lambda x\left(1-\frac{x}{K}\right)$ describes the logistic growth rate of GSCs;
- s is the normalized rate of SOX2 expression level in a GSC, which ranges between 0 and 1;
- The constant value α represents the strength of infectivity of the Zika virus in the GSCs;
- The term $\alpha sx\left(t\right)v\left(t\right)$ describes the rate of infected cells by free virus, $v\left(t\right)$;
- b is the bursting size of free virus particles;
- $\delta_{1}$ represents the death rate of infected GSCs after the cell oncolysis;
- $\delta_{2}$ is the clearance rate of the virus.

For non-dimensionalization, we set $\tau =\delta_{1}t$, $x=K\tilde{x}$, $y=K\tilde{y}$, and $v=K\tilde{v}$. Then,

(2) $$\frac{\text{dτ}}{\text{dt}}={\text{δ}}_{1},\;\frac{\text{dx}}{\text{dt}}={\text{δ}}_{1}\text{K}\frac{\text{d}\tilde{\text{x}}}{\text{dτ}},\;\frac{\text{dy}}{\text{dt}}={\text{δ}}_{1}\text{K}\frac{\text{d}\tilde{\text{y}}}{\text{dτ}},\;\frac{\text{dv}}{\text{dt}}={\text{δ}}_{1}\text{K}\frac{\text{d}\tilde{\text{v}}}{\text{dτ}}$$

The system of Equation (1) becomes

(3) $$\frac{\text{d}\tilde{\text{x}}}{\text{dτ}}=\frac{\text{λ}}{{\text{δ}}_{1}}\tilde{\text{x}}\left(1-\tilde{\text{x}}\right)-\frac{\text{αsK}}{{\text{δ}}_{1}}\tilde{\text{x}}\tilde{\text{v}}\;\frac{\text{d}\tilde{\text{y}}}{\text{dτ}}=\frac{\text{αsK}}{{\text{δ}}_{1}}\tilde{\text{x}}\tilde{\text{v}}-\tilde{\text{y}}\;\frac{\text{d}\tilde{\text{v}}}{\text{dτ}}=\text{b}\tilde{\text{y}}-\frac{\text{αsK}}{{\text{δ}}_{1}}\tilde{\text{x}}\tilde{\text{v}}-\frac{{\text{δ}}_{2}}{{\text{δ}}_{1}}\tilde{\text{v}}$$

We have the following model by setting the parameters:

$r=\frac{\lambda }{\delta_{1}}$, $a=\frac{\alpha K}{\delta_{1}}$ and $\delta_{v}=\frac{\delta_{2}}{\delta_{1}}$. For convenience, we write $\tilde{x}=x$, $\tilde{y}=y$, $\tilde{v}=v$, and τ=t.

Then the system of Equation (3) becomes

(4) $$\frac{dx\left(t\right)}{dt}=rx\left(t\right)\left(1-x\left(t\right)\right)-asx\left(t\right)v\left(t\right)\;\frac{dy\left(t\right)}{dt}=asx\left(t\right)v\left(t\right)-y\left(t\right)\;\frac{dv\left(t\right)}{dt}=by\left(t\right)-asx\left(t\right)v\left(t\right)-\delta_{v}v\left(t\right)$$

Note that all the parameters (r, a, s, b, and $\delta_{v}$) in the system of Equation (4) are positive constants.

**Theorem 1.** If $x\left(0\right)\geq 0$, $y\left(0\right)\geq 0$, and $v\left(0\right)\geq 0$, then $x\left(t\right)\geq 0$, $y\left(t\right)\geq 0$, and $v\left(t\right)\geq 0$ for t≥0. Furthermore, the component $x\left(t\right)$ is bounded and belongs to the interval [0, 1] for all t≥0.

**Proof.** If the conclusion $x\left(t\right)\geq 0$, $y\left(t\right)\geq 0$, and $v\left(t\right)\geq 0$ for t≥0 is not true, then there exists a time $t=t^{*}$ at which at least one component first becomes zero. We examine each possible case.

Case 1: If the one component is zero at $t=t^{*}$.

1. If $x\left(t^{*}\right)=0$, then $x^{\prime }\left(t^{*}\right)=0$. From the first equation of the system of Equation (4), $x\left(t\right)=0$ for all $t\geq t^{*}$ by the uniqueness of the solution. Then, the second equation of the system of Equation (4) becomes $y^{\prime }\left(t\right)=-y$. Using the separation of variable method, $y\left(t\right)=y\left(t^{*}\right)e^{-t+t^{*}}$. This implies $y\left(t\right)\geq 0$, since $y\left(t^{*}\right)\geq 0$. From the third equation of the system of Equation (4), we have $v^{\prime }\left(t\right)=by-\delta_{v}v$. This is a non-homogeneous linear differential equation because of the term *by*. The homogeneous solution is $v_{h}\left(t\right)=v\left(t^{*}\right)e^{-\delta_{v}\left(t-t^{*}\right)}$ and the particular solution is $v_{p}\left(t\right)=be^{-\delta_{v}\left(t-t^{*}\right)}\int_{t^{*}}^{t}e^{\delta_{v}u}y\left(u\right)du$. The general solution is $v\left(t\right)=v_{h}\left(t\right)+v_{p}\left(t\right)=v\left(t^{*}\right)e^{-\delta_{v}\left(t-t^{*}\right)}+be^{-\delta_{v}\left(t-t^{*}\right)}\int_{t^{*}}^{t}e^{\delta_{v}u}y\left(u\right)du$. Therefore, $v\left(t\right)\geq 0$.
2. If $y\left(t^{\text{*}}\right)=0$, then $y^{\prime }\left(t^{\text{*}}\right)=asx\left(t^{\text{*}}\right)v\left(t^{\text{*}}\right)$. The first equation of the system of equation (4) is $\frac{dx}{x}=\left(r\left(1-x\right)-asv\right)dt$. Using the separation of variable method over $\left[t^{*},t\right]$, $x\left(t\right)=x\left(t^{*}\right)e^{\int_{t^{*}}^{t}r\left(1-x\right)-asv\;du}$. So, $x\left(t\right)>0$ for all $t\geq t^{\text{*}}$. From the third equation of the system of Equation (4), we have $v^{\prime }\left(t\right)=-asxv-\delta_{v}v\Leftrightarrow \frac{1}{v}dt=\left(-asx-\delta_{v}\right)dt$. Similarly, $v\left(t\right)=v(t^{\text{*}})e^{-\int_{t^{\text{*}}}^{t}asx\left(u\right)+\delta_{v}\;du}$. Thus, $v\left(t\right)>0\;\cdot t\geq t^{*}$. Therefore, $y\left(t\right)\geq 0$ when $t\geq t^{*}$.
3. If $v\left(t^{\text{*}}\right)=0$, then $v^{\prime }\left(t^{\text{*}}\right)=by\left(t^{\text{*}}\right)\geq 0$. So, $v\left(t\right)\geq 0$ for $t\geq t^{*}$, since $x\left(t\right)=\frac{x\left(t^{*}\right)}{x\left(t^{*}\right)+\left(1-x\left(t^{*}\right)\right)e^{-r\left(t-t^{*}\right)}}>0$, $y\left(t\right)=y\left(t^{*}\right)e^{-\left(t-t^{*}\right)}>0$ for all $t\geq t^{*}$.

Case 2: If the two components are zero simultaneously at $t=t^{*}$, it is easy to show that the third component will be non-negative for all $t\geq t^{*}$.

Case 3: If the three components are zero simultaneously at $t=t^{*}$, then $x\left(t\right)=0$, $y\left(t\right)=0$, and $v\left(t\right)=0$ for all $t\geq t^{*}$ by the uniqueness of the solution.

Therefore, $x\left(t\right)\geq 0$, $y\left(t\right)\geq 0$, and $v\left(t\right)\geq 0$ for all t≥0. □

For boundness, from the first equation of the system of Equation (4), $\frac{dx\left(t\right)}{dt}=rx\left(t\right)\left(1-x\left(t\right)\right)-asx\left(t\right)v\left(t\right)\leq rx\left(t\right)\left(1-x\left(t\right)\right)$. Let $\frac{dX}{dt}=rX\left(t\right)\left(1-X\left(t\right)\right)$, with initial condition $X\left(0\right)=X_{0}$, then the solution of the differential equation can be obtained by the separation of variable method and is given by $X\left(t\right)=\frac{X_{0}}{X_{0}+\left(1-X_{0}\right)e^{-rt}}$. Since $\frac{dx}{dt}\leq \frac{dX}{dt}$ and $\text{li}{\text{m}}_{t\to \infty }supX\left(t\right)=1$, we have $\text{li}{\text{m}}_{t\to \infty }sup\;x\left(t\right)\leq \text{li}{\text{m}}_{t\to \infty }sup\;X\left(t\right)=1$.

Therefore, all the solutions of the system of Equation (4) are non-negative and $\text{x}\left(\text{t}\right)$ is bounded in the following region:

$$D=\text{\{}\left(x,y,v\right)\in R^{3}|0\leq x\leq 1,y\geq 0,v\geq 0\}.$$

D is the positive invariant where every solution with initial condition in D remains there for all t≥0.

### 2.2. Analysis and Stability of Equilibrium

The equilibrium points of the system are obtained by setting the right-hand side of the system of Equation (4) to zero. Let $X={\left(x,\;y,\;v\right)}^{T}$ and $F\left(x\right)={\left(rx\left(1-x\right)-asxv,\;asxv-y,\;by-asxv-\delta_{v}v\right)}^{T}$.

Then, the system can be written as the autonomous system $\frac{dX}{dt}=F\left(X\right)$. We assume that the solution set $\left(x,y,v\right)$ of the system of Equation (4) are in D. Simply, the equilibrium points are the solution of $\frac{dX}{dt}=0$ or $F\left(X\right)=0$ which is given by

(5) $$\begin{array}{c} rx\left(1-x\right)-asxv=0,\\ asxv-y=0,\\ by-asxv-\delta_{v}=0, \end{array}$$

1. If x=0, then y=0 and v=0 from the second and the third equations in Equation (5). Therefore, we have an equilibrium point $E_{0}\left(0,0,0\right)$.
2. If x≠0 and v=0, we get y=0 from the second equation, which results in $r\left(1-x\right)=0$ from the first equation in Equation (5). Then, x=1. Thus, we have an equilibrium point $E_{1}\left(1,0,0\right)$.
3. If x≠0 and v≠0, then, from the second and third equations in Equation (5), $absxv-asxv-\delta_{v}v=0\Rightarrow v\left(absx-asx-\delta_{v}\right)=0$. Since v≠0, we have $absx-asx-\delta_{v}=0\Rightarrow x=\frac{\delta_{v}}{as\left(b-1\right)}$. From the first and second equation in Equation (5), $rx\left(1-x\right)-y=0\Rightarrow y=rx\left(1-x\right)$. Then, $y=\frac{r\delta_{v}}{as\left(b-1\right)}\left(1-\frac{\delta_{v}}{as\left(b-1\right)}\right)$. From the third equation in Equation (5), $by-y-\delta_{v}v=0\Rightarrow v=\frac{\left(b-1\right)}{\delta_{v}}y$. Then, $v=\frac{r}{as\delta_{v}}\left(1-\frac{\delta_{v}}{as\left(b-1\right)}\right)$. Thus, we have an equilibrium point $E_{2}\left(x_{2},\;y_{2},\;v_{2}\right)$, where $x_{2}=\frac{\delta_{v}}{as\left(b-1\right)}$, $y_{2}=\frac{r\delta_{v}}{as\left(b-1\right)}\left(1-\frac{\delta_{v}}{as\left(b-1\right)}\right)$, and $v_{2}=\frac{r}{as}\left(1-\frac{\delta_{v}}{as\left(b-1\right)}\right)$.

### 2.3. Basic Reproduction Number

We consider the basic reproduction number as the number of secondary cases of infection generated from a single Zika virus in a GSC population where all GSCs are susceptible to infection. We utilize the next-generation matrix technique to calculate the basic reproductive number for the system of Equation (4).

Let $\Phi =\left(x,y,v\right)$, then all three equations of the system of Equation (4) can be written as $\Phi^{\prime }=A\left(\Phi \right)-B\left(\Phi \right)$, where

$$A\left(\text{Φ}\right)=\left(\begin{array}{c} 0\\ asxv\\ 0 \end{array}\right)\text{ and }B\left(\text{Φ}\right)=\left(\begin{array}{c} -rx\left(1-x\right)+asxv\\ y\\ -by+asxv+\delta_{v}v \end{array}\right)$$

The Jacobian matrix of A and B at $E_{1}\left(1,0,0\right)$ is

$$J\left(A\left(E_{1}\right)\right)=\left(\begin{array}{ccc} 0 & 0 & 0\\ 0 & 0 & as\\ 0 & 0 & 0 \end{array}\right),\;J\left(B\left(E_{1}\right)\right)=\left(\begin{array}{ccc} r & 0 & as\\ 0 & 1 & 0\\ 0 & -b & as+\delta_{v} \end{array}\right)$$

The basic reproduction number is obtained from the eigenvalue of $J\left(A\left(E_{1}\right)\right)\;J{\left(B\left(E_{1}\right)\right)}^{-1}$, so we have

$$R_{0}=\frac{abs}{as+\delta_{v}}$$

### 2.4. Stability of Equilibrium Points

We studied the local stability of the equilibrium points using the linear stability analysis by finding the eigenvalues of Jacobian matrix at each equilibrium point. The Jacobian matrix of the nonlinear system in Equation (5) is given by

$$J\left(x,y,v\right)=\left(\begin{array}{ccc} r-2rx-asv & 0 & -asv\\ asv & -1 & asx\\ -asv & b & -asx-\delta_{v} \end{array}\right)$$

**Theorem 2.** The equilibrium point $E_{0}\left(0,0,0\right)$ is always unstable.

**Proof.** The Jacobian matrix at $E_{0}\left(0,0,0\right)$ is

$$J\left(E_{0}\right)=\left(\begin{array}{c} r\\ 0\\ 0 \end{array}\;\;\begin{array}{c} 0\\ -1\\ b \end{array}\;\;\begin{array}{c} 0\\ 0\\ -\delta_{v} \end{array}\right)$$

The eigenvalues are $\lambda_{1}=r$, $\lambda_{2}=-1$ and $\lambda_{3}=\;-\delta_{v}$. All parameters are positive values. $\lambda_{2}$ and $\lambda_{3}$ are negative but $\lambda_{1}$ is positive. Therefore, the equilibrium point $E_{0}\left(0,0,0\right)$ is unstable. □

**Theorem 3.** The equilibrium point $E_{1}\left(1,0,0\right)$ is locally asymptotically stable if $R_{0}<1$. Otherwise, it is unstable.

**Proof.** The Jacobian matrix at $E_{1}\left(1,0,0\right)$ is given by

$$J\left(E_{1}\right)=\left(\begin{array}{ccc} -r & 0 & -as\\ 0 & -1 & as\\ 0 & b & -as-\delta_{v} \end{array}\right)$$

Solving the characteristic equation, $\left|J\left(E_{1}\right)-\lambda I\right|=0$, we have $\lambda_{1}=-r<0$ since r>0 and $\lambda_{2}$ and $\lambda_{3}$ satisfy the equation $\lambda^{2}+a_{1}\lambda +a_{2}=0$, where $a_{1}=1+as+\delta_{v}$ and $a_{2}=as+\delta_{v}-abs$. By Routh–Hurwitz criteria [42], the eigenvalues $\lambda_{2}$ and $\lambda_{3}$ are negative if $a_{1}>0$ and $a_{2}>0$. Clearly, $a_{1}>0$ since all parameters are positive and for $a_{2}$ to be positive, $a_{2}=as+\delta_{v}-abs>0$, which implies $R_{0}=\frac{abs}{as+\delta_{v}}<1$. Therefore, if $R_{0}<1$, $E_{1}\left(1,\;0,\;0\right)$ is asymptotically stable. Otherwise, $E_{1}\left(1,\;0,\;0\right)$ becomes unstable. Furthermore, $R_{0}=\frac{abs}{as+\delta_{v}}<1\Leftrightarrow as\left(b-1\right)<\delta_{v}\Leftrightarrow b-1<\frac{\delta_{v}}{as}\Leftrightarrow b<\frac{\delta_{v}}{as}+1$, which leads b<1. □

The equilibrium point $E_{1}\left(1,\;0,\;0\right)$ represents a scenario where the population of GSC is at the carrying capacity (or maximum value of 1), indicating no infection has taken place. This means that the virotherapy has failed completely, as all GSCs remain uninfected. The theorem states that $E_{1}\left(1,\;0,\;0\right)$ is locally asymptotically stable if $R_{0}<1$. This implies that when the basic reproduction number $R_{0}$ is less than 1, each infected cell generates, on average, less than one new infected cell, leading to the decline and eventual extinction of the infection. As a result, the therapy is infective and the GSCs remain intact. Conversely, if $R_{0}$ is greater than or equal to 1, the infection can spread, making $E_{1}\left(1,\;0,\;0\right)$ unstable.

**Theorem 4.** The equilibrium point $E_{2}\left(x_{2},y_{2},v_{2}\right)$ is locally asymptotically stable if b>1 and $R_{0}>1$.

**Proof.** The Jacobian matrix at $E_{2}\left(x_{2},y_{2},v_{2}\right)$ is

$$J\left(E_{2}\right)=\left(\begin{array}{ccc} r-2rx_{2}-asv_{2} & 0 & -asx_{2}\\ asv_{2} & -1 & asx_{2}\\ -asv_{2} & b & -asx_{2}-\delta_{v} \end{array}\right)$$

Since $r-rx_{2}-asv_{2}=0$ from the first equation in the system of Equation (5),

$$J\left(E_{2}\right)=\left(\begin{array}{ccc} -rx_{2} & 0 & -asx_{2}\\ asv_{2} & -1 & asx_{2}\\ -asv_{2} & b & -asx_{2}-\delta_{v} \end{array}\right)$$

The characteristic equation is $\lambda^{3}+a_{1}\lambda^{2}+a_{2}\lambda +a_{3}=0$, □

where $a_{1}=asx_{2}+rx_{2}+\delta_{v}+1$, $a_{2}=asx_{2}^{2}+asx_{2}+r\delta_{v}x_{2}+rx_{2}+\delta_{v}+arsx_{2}v_{2}-a^{2}s^{2}x_{2}v_{2}-absx_{2}$, and $a_{3}=a^{2}bs^{2}x_{2}v_{2}+ars\delta_{v}x_{2}v_{2}+arsx_{2}^{2}+r\delta_{v}x_{2}-a^{2}s^{2}x_{2}v_{2}-abrsx_{2}^{2}$. Therefore, we have $a_{1}=\frac{as\left(b\delta_{v}+b-1\right)+r\delta_{v}}{as\left(b-1\right)}$, $a_{2}=\frac{\delta_{v}(as\left(b-1\right)\left(\left(b-1\right)r^{2}+\left(\delta_{v}+1\right)\left(b-1\right)r+\delta_{v}\right)+r\delta_{v}^{2}\left(1+r\left(b-1\right))\right)}{as{\left(b-1\right)}^{2}\left(as\left(b-1\right)+r\delta_{v}\right)}$, and $a_{3}=\frac{r\delta_{v}\left(as\left(b-1\right)-\delta_{v}\right)}{as\left(b-1\right)}$. It is clear that $a_{1}>0$ and $a_{3}>0$ since the equilibrium point $E_{2}\left(x_{2},y_{2},v_{2}\right)$ exists if b>1 and $a>\frac{\delta_{v}\;}{s\left(b-1\right)}\Leftrightarrow as\left(b-1\right)-\delta_{v}>0$. It is enough to show that $a_{1}\cdot a_{2}>a_{3}$ to prove that the eigenvalues of $\lambda^{3}+a_{1}\lambda^{2}+a_{2}\lambda +a_{3}=0$ are negative by Routh–Hurwitz criteria.


$$a_{1}\cdot a_{2}-a_{3}=\frac{as\left(b\delta_{v}+b-1\right)+r\delta_{v}}{as\left(b-1\right)}\cdot \frac{\delta_{v}\left[as\left(b-1\right)\left(\left(b-1\right)r^{2}+\left(\delta_{v}+1\right)\left(b-1\right)r+\delta_{v}\right)+r\delta_{v}^{2}\left(1+r\left(b-1\right)\right)\right]}{as{\left(b-1\right)}^{2}\left(as\left(b-1\right)+r\delta_{v}\right)}-\frac{r\delta_{v}\left(as\left(b-1\right)-\delta_{v}\right)}{as\left(b-1\right)}$$

$$=\frac{r\left(as\left(b\delta_{v}+b-1\right)+r\delta_{v}\right)}{as\left(b-1\right)}\cdot \frac{b^{2}-1+\delta_{v}+r\delta_{v}\left(1+r\left(b-1\right)\right)}{\delta_{v}{\left(as\left(b-1\right)\right)}^{2}\left(as\left(b-1\right)+r\delta_{v}\right)}$$

$$=\frac{r\left(as\left(b\delta_{v}+b-1\right)+r\delta_{v}\right)}{as\left(b-1\right)}\cdot \frac{\left(b+1\right)\left(b-1\right)+\delta_{v}+r\delta_{v}\left(1+r\left(b-1\right)\right)}{\delta_{v}{\left(as\left(b-1\right)\right)}^{2}\left(as\left(b-1\right)+r\delta_{v}\right)}$$

$$=\frac{r\left(as\left(b\delta_{v}+b-1\right)+r\delta_{v}\right)}{as\left(b-1\right)}\cdot \frac{\left(b+1\right)(b-1+r\delta_{v}\left(1+r\left(b-1\right)\right)+\delta_{v}}{\delta_{v}{\left(as\left(b-1\right)\right)}^{2}\left(as\left(b-1\right)+r\delta_{v}\right)}.$$

Now, the term $\left(b+1\right)(b-1+r\delta_{v}\left(1+r\left(b-1\right)\right)+\delta_{v}$ is always positive, so we can ignore it when considering the sign of $a_{1}\cdot a_{2}-a_{3}$. Therefore, $a_{1}\cdot a_{2}-a_{3}>0\Leftrightarrow \frac{r\left(as\left(b\delta_{v}+b-1\right)+r\delta_{v}\right)}{as\left(b-1\right)}\cdot \frac{1}{\delta_{v}{\left(as\left(b-1\right)\right)}^{2}\left(as\left(b-1\right)+r\delta_{v}\right)}>0$ holds if and only if $\frac{r\left(as\left(b\delta_{v}+b-1\right)+r\delta_{v}\right)}{as\left(b-1\right)}>0$. This inequality is equivalent to $r\left(as\left(b\delta_{v}+b-1\right)+r\delta_{v}\right)>0$. Therefore, we have shown that $a_{1}\cdot a_{2}-a_{3}>0$ if and only if $R_{0}>1$. Note that $R_{0}=\frac{abs}{as+\delta_{v}}>1\Leftrightarrow as\left(b-1\right)>\delta_{v}\Leftrightarrow b-1>\frac{\delta_{v}}{as}\Leftrightarrow b>\frac{\delta_{v}}{as}+1=\frac{as+\delta_{v}}{as}>1\Leftrightarrow b>1$.

Therefore, from Theorems 3 and 4, the stability of $E_{1}$ and $E_{2}$ changes about b=1, which means a transcriptical bifurcation happens at b=1.

The equilibrium point $E_{2}\left(x_{2},y_{2},v_{2}\right)$ corresponds to a situation where there is a balance between the populations of GSCs, infected GSCs, and the ZIKV. This balance indicates that the infection is present and affects GSCs. The theorem states that $E_{2}\left(x_{2},y_{2},v_{2}\right)$ is locally asymptotically stable if $R_{0}>1$. This implies that when the basic reproduction number $R_{0}$ is greater than 1, each infected GSC generates more than one new infected GSC, leading to the sustained spread of the infection. In this scenario, the ZIKV effectively infects the GSCs and reduces their population indicating successful virotherapy.

### 2.5. Estimation of Parameters

Parameters were estimated from the experimental data (Table 1) [28]. In the experiment, there were 1 million cancer stem cells on day 0, and the number of cancer stem cells increased to 9 million by day 7. The difference in cell count from day 0 to day 7 was 8 million cells, and the population followed exponential growth. $8\text{ million}=e^{\lambda *7}\to \lambda =\frac{\text{ln}\left(8\right)}{7}=0.2971\text{ million cells/day}$. In the experiment [28], they infected GSCs with ZIKV at a multiplicity of infection (MOI) of 5. More than 60% of GSCs were infected 48 h after infection, and 90% of the infected cells expressed SOX2. We estimated the infection rate as follows: the initial number of GSCs was 1 million, and the MOI was 5. Thus, the number of infectious viral particles was 1 million × 5=5 million. Therefore, the number of infected cells at 48 h was 60% × 5 millions=3 million. Given that 90% of the infected cells expressed SOX2, the number of SOX2-expressing infected cells was 90% × 3 million = 2.7 million. The infection rate can be estimated as the fraction of infected cells produced per unit time per initial number of cells. Assuming exponential growth of infection over time, we estimated the average rate of increase in the number of infected cells. Since we observed 3 million infected cells at 48 h, we can estimate the average rate of increase over the first 48 h. Therefore, the infection rate would be 2.7 million/(1 million × 5× 2 days) = 0.054 per day. The ZIKVs were cleaned up after day 8, so the death rate of ZIKV was 1/8 = 0.125 per day.

### 2.6. Sensitivity Analysis

Sensitivity analysis is applied to study the effect of parameters on the proposed mathematical model. In particular, it is necessary to identify the most sensitive parameters that cause a disturbance in the model dynamics with a small change in their numeric values. To check the sensitivity of $R_{0}$, sensitivity, we calculate its derivatives as follows:

$$\frac{\partial R_{0}}{\partial a}=\frac{bs\delta_{v}}{{\left(as+\delta_{v}\right)}^{2}},\;\frac{\partial R_{0}}{\partial b}=\frac{as}{as+\delta_{v}},\;\frac{\partial R_{0}}{\partial \delta_{v}}=-\frac{abs}{{\left(as+\delta_{v}\right)}^{2}},\text{and}\frac{\partial R_{0}}{\partial s}=\frac{ab\delta_{v}}{{\left(as+\delta_{v}\right)}^{2}}$$

Since all the parameters are positive, $\frac{\partial R_{0}}{\partial a}$, $\frac{\partial R_{0}}{\partial b}$, and $\frac{\partial R_{0}}{\partial s}>0$. It concludes that the basic reproduction number $R_{0}$ increases as a, b and s increase. The normalized sensitivity indices corresponding to these parameters are estimated as follows:

$$\Gamma_{a}=\frac{a}{R_{0}}\frac{\partial R_{0}}{\partial a}=\frac{\delta_{v}}{as+\delta_{v}},\;\Gamma_{b}=\frac{b}{R_{0}}\frac{\partial R_{0}}{\partial b}=1,\;\Gamma_{\delta_{v}}=\frac{\delta_{v}}{R_{0}}\frac{\partial R_{0}}{\partial \delta_{v}}=-\frac{\delta_{v}}{as+\delta_{v}},\text{and }\Gamma_{s}=\frac{s}{R_{0}}\frac{\partial R_{0}}{\partial s}=\frac{\delta_{v}}{as+\delta_{v}}$$

**Figure 1.**
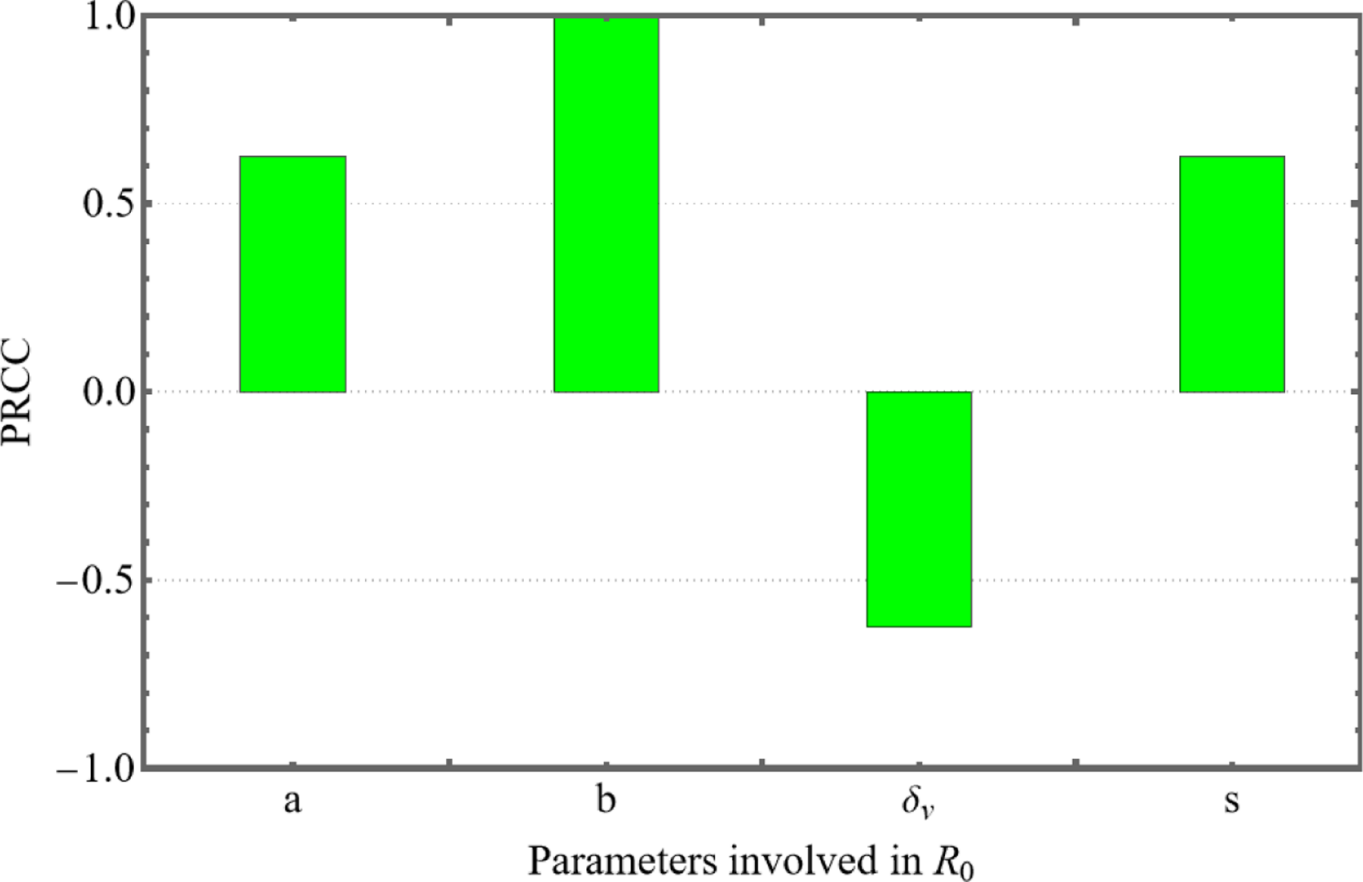
PRCC results for significance of parameters involved in $R_{0}$.

Here, the sensitivity index can be constant depending on some parameters or can be free of any independent parameters. The partial rank correlation coefficient (PRCC) results for significance of parameters involved in $R_{0}$ is shown in Figure 1. The positive PRCC values for a, b, and s indicate that these parameters are directly correlated with $R_{0}$, meaning that increases in these parameters lead to higher $R_{0}$ values, thereby enhancing the infection spread. On the other hand, the negative PRCC value for $\delta_{v}$ demonstrates its inverse relationship with $R_{0}$; as $\delta_{v}$ increases, $R_{0}$ decreases, suggesting that enhancing virus clearance is crucial for controlling the infection. This sensitivity analysis emphasizes the importance of accurately estimating these parameters, as small changes in their values can significantly alter the infection dynamics, guiding effective intervention strategies.

## 3. Results

The nondimensionalized model (Equation (4)) was employed to present the numerical results. For these calculations, we utilized the Runge–Kutta 2nd order method with a time step of Δt=0.05 in MATLAB R2023a (The MathWorks, Natick, MA, USA). To assess the accuracy of the numerical scheme, we also tested smaller values of Δt and compared the results with those obtained using the Runge–Kutta 4th order method.

### 3.1. Existence and Stability of the Equilibrium Points

**Figure 2.**
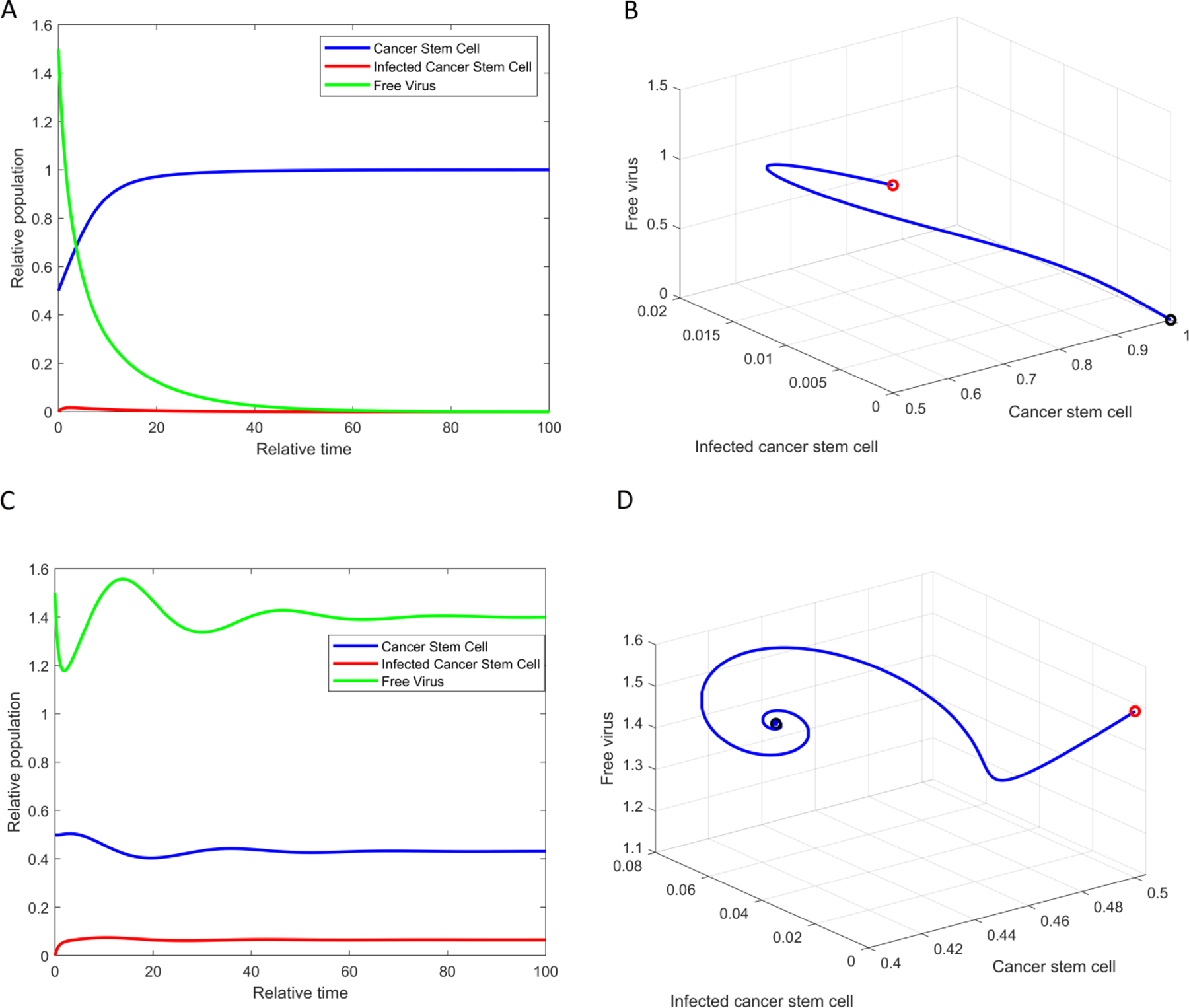
The relative population solution of GSCs, infected GSCs, and ZIKV. Population dynamics over time (**A**) and in phase space (**B**) when the SOX2 expression level constant s=0.3 and $R_{0}<1$, showing the equilibrium point $E_{1}\left(1,0,0\right)$ is asymptotically stable. (**C**,**D**) are the case when s=1 and $R_{0}>1$, ensuring the equilibrium point $E_{2}\left(x_{2},y_{2},v_{2}\right)$ becomes stable. The red circle represents the initial condition, and the black circle indicates the equilibrium point in (**B**,**D**). We used parameters r=0.3, a=0.108, $\delta_{v}=0.3254$, and b=8. We also used initial conditions $x\left(0\right)=0.5$, $y\left(0\right)=0$, and $v\left(0\right)=1.5$.

For numerical simulations, we initially set parameters as r=0.3, a=0.108, $\delta_{v}=0.3254$, and b=8. However, we may adjust certain parameter values to ensure that the existence and stability conditions for each equilibrium point are met. Figure 2 illustrates the population solutions over relative time (A and C) and the trajectories of solutions in the phase space (B and D). We set the SOX2 expression level constant to the value s=0.3, which satisfies the condition $R_{0}=\frac{abs}{as+\delta_{v}}=0.7244<1$, ensuring that $E_{1}\left(1,0,0\right)$ becomes asymptotically stable (Figure 2A,B). The equilibrium point $E_{2}\left(x_{2},y_{2},v_{2}\right)$ becomes asymptotically stable when s=1, while $E_{1}\left(1,0,0\right)$ becomes asymptotically unstable and it satisfies the condition $R_{0}=1.9935>1$. The solution $\left(x\left(t\right),y\left(t\right),v\left(t\right)\right)$ converges to $\left(0.4304,0.0651,1.4012\right)$. This result provides a good agreement between our analytic and numerical results.

### 3.2. SOX2 Expression Level Changes the Structure of GSC Dynamics

**Figure 3.**
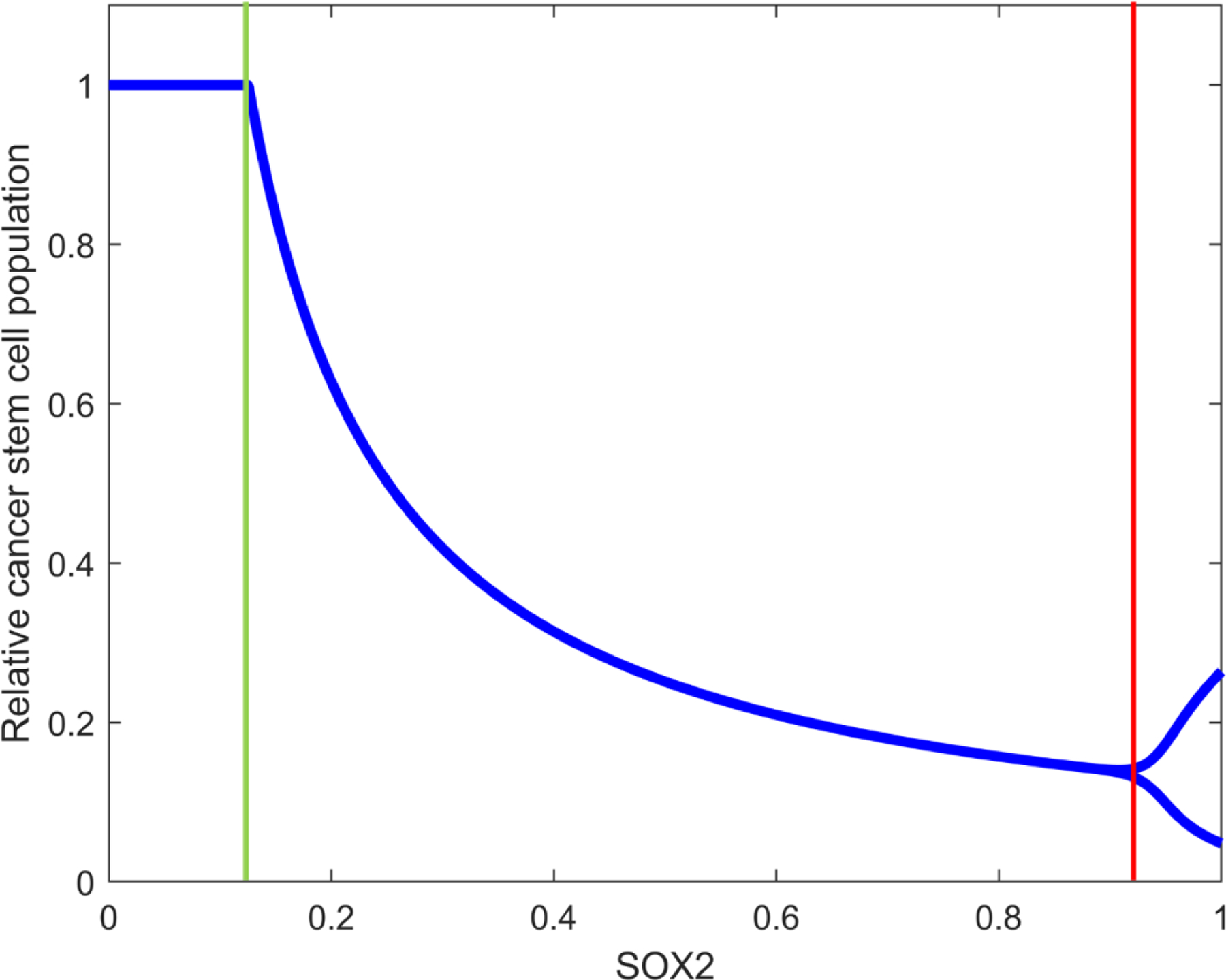
Bifurcation diagram of the GSC population with respect to the bifurcation parameter SOX2 expression level constant $\left(s\right)$. The figure illustrates two bifurcation thresholds at $s=s_{1}^{*}=0.17$ (green vertical line), where the transcritical bifurcation occurs, and at $s=s_{2}^{*}=0.93$ (red vertical line), where the Hopf bifurcation occurs. We used the parameters r=0.3, a=0.108, $\delta_{v}=0.3254$, and b=25 with initial conditions $x\left(0\right)=0.5$, $y\left(0\right)=0$, and $v\left(0\right)=1.5$.

The bifurcation diagram of GSCs with respect to the normalized SOX2 expression levels is shown in Figure 3. In this simulation, we set the virus bursting size b=25. Our numerical result shows two bifurcation values: (1) A transcritical bifurcation occurs at $s=s_{1}^{*}=0.17$ (green colored vertical line in Figure 3) which results in a qualitative change in stability between equilibrium points $E_{1}\left(1,\;0,\;0\right)$ and $E_{2}\left(x_{2},y_{2},v_{2}\right)$. When $s<s_{1}^{*}$, $E_{1}$ is stable, while $E_{2}$ is unstable. However, the stability of two equilibrium points changes for $s>s_{1}^{*}$; $E_{2}$ becomes stable and $E_{1}$ becomes unstable. (2) A Hopf bifurcation is observed at $s=s_{2}^{*}=0.93$ (red colored vertical line in Figure 3), which leads to oscillatory behavior in populations. When $s<s_{2}^{*}$, $E_{2}$ is a stable equilibrium point. However, when $s>s_{2}^{*}$, the system undergoes a qualitative change in dynamics such as a periodic cycle around $E_{2}$. This result explains the sensitivity of the system to SOX2 expression level, with s=0.93 which represents a critical value where periodic patterns become prominent in the GSC–ZIKV dynamical system.

From a biological point of view, the dynamic interplay among GSCs, infected GSCs, and ZIKV has direct implications for therapeutic efficacy. For example, equilibrium point $E_{1}$ represents a free virus equilibrium point where therapy fails (GSC population approaches to the carrying capacity). The other equilibrium point $E_{2}$ represents partial success which indicates a reduction in the GSC population. This bifurcation analysis provides a good understanding of the complex population dynamics within the GSC–ZIKV system. These results suggest a critical threshold of SOX2 expression level is an important factor to be considered for therapeutic strategies to eradicate the GSCs in the tumor microenvironment.

### 3.3. Interplay between SOX2 Expression Level and Bursting Size Affects the Dynamic of OVT

**Figure 4.**
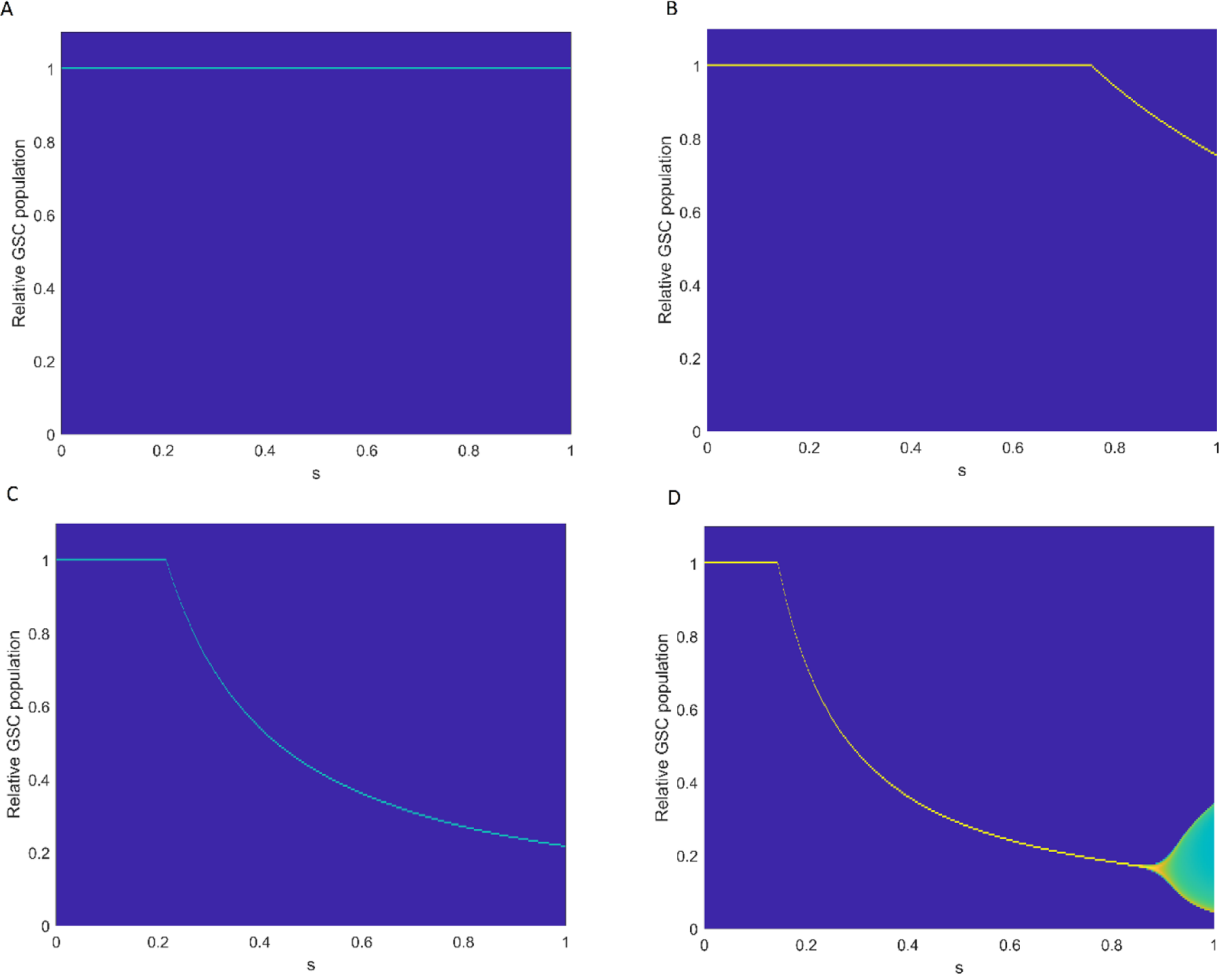
Two-dimensional bifurcation diagram of GSC population with respect to the bifurcation parameter SOX2 expression level constant $\left(s\right)$ with different values of bursting rate. (**A**–**D**) Relative GSC populations over SOX2 expression level constant when b=4, 5, 15, and 20, respectively. We used the parameters r=0.3, a=0.108, $\delta_{v}=0.3254$ with initial conditions $x\left(0\right)=0.5$, $y\left(0\right)=0$, and $v\left(0\right)=1.5$.

For different values of the bursting size (b), we investigated the effect of SOX2 expression level (s) on GSC dynamics with different values of the bursting size value b shown in Figure 4 (A–D were when b=4, 5, 15, and 20, respectively). For b≤4, the GSC population converged to the carrying capacity, which resulted in the therapy failure. For b=5, a transcritical bifurcation occurred at $s=s^{*}=0.75$ where $E_{2}$ became stable while $E_{1}$ became unstable. The GSC population reached its minimum of 0.78 at s=1. Increasing the bursting size to 15 resulted in shifting a transcritical bifurcation threshold to $s=s^{*}=0.23$. The minimum GSC population, 0.23, was observed at s=1. Finally, for b=20, there were two bifurcations threshold values: a transcritical bifurcation at $s^{*}=0.14$ and a Hopf bifurcation at $s^{**}=0.9$. At $s=s^{**}=0.9$, the minimum population was 0.167. Moreover, when $s>s^{**}$, all populations showed oscillations. These results indicate a shift in the transcritical bifurcation point $s=s^{*}$ to a lower value of the bursting size b≥5 and the induction of an oscillatory pattern with higher bursting size.

Both SOX2 expression level (s) and virus bursting size (b) play an important role in OVT since (1) SOX2 expression levels activate the membrane receptor αvβ5, which results in an enhancing of the infection rate of ZIKV into GSCs and (2) the virus bursting size is directly proportional to the basic reproduction number $R_{0}$, which can change the stability of equilibrium points and affect the efficacy of the therapy. Understanding the underlying mechanisms of changes of stability of equilibrium points or of structure of dynamics (or bifurcation) helps to show the importance of the optimization of parameters in the system and in treatment efficacy.

### 3.4. Stability Regions for Equilibrium Points in Two-Dimensional Parameters: SOX2 Expression Level and Bursting Size

**Figure 5.**
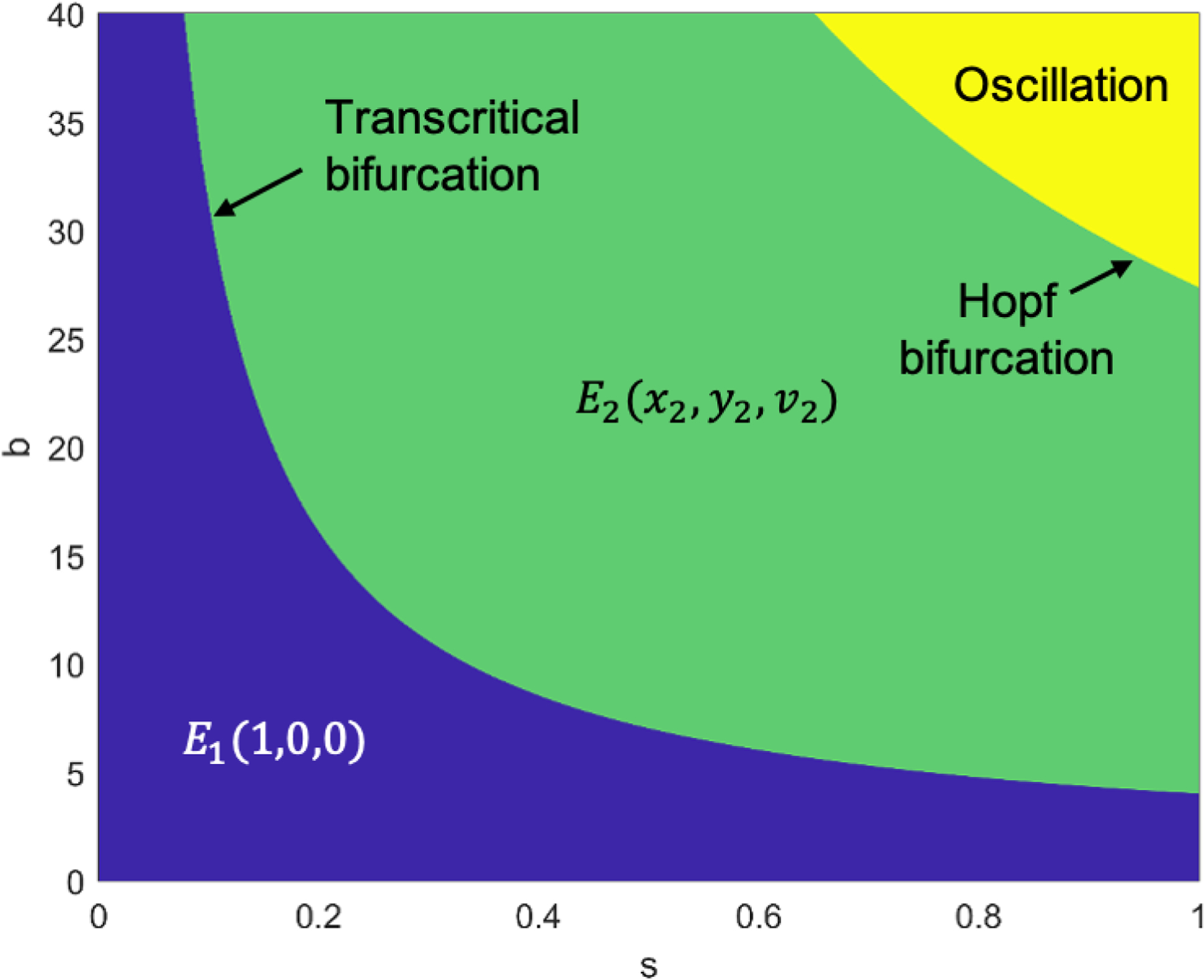
Stability region of equilibrium points with respect to two parameters (b and s). The equilibrium point $E_{1}\left(1,0,0\right)$ is asymptotically stable in the blue region, while the equilibrium point $E_{2}\left(x_{2},y_{2},v_{2}\right)$ is asymptotically stable in the green region, and three populations (GSCs, infected GSCs, and ZIKV) oscillate over time in the yellow region. We used the parameters r=0.3, a=0.108, $\delta_{v}=0.3254$, with initial conditions $x\left(0\right)=0.5$, $y\left(0\right)=0$, and $v\left(0\right)=1.5$.

In our mathematical exploration, we systematically varied the bursting size (b) and SOX2 expression level (s), conducting simulations to calculate eigenvalues and visualize stability regions for different equilibrium points on the s-b coordinate. The two-dimensional bifurcation diagram is shown in Figure 5. The color-coded representation denotes blue for the stability region of equilibrium point $E_{1}$, green for $E_{2}$ stability, and yellow for regions where oscillations occur. The boundary between the green and yellow regions highlights the minimum GSC population. These stability regions elucidate critical points in the interplay between SOX2 expression, bursting size, and GSC dynamics. Transitions among the blue, green, and yellow regions signify qualitative shifts, reflecting the impact of SOX2 expression levels on GSCs. Understanding these stability regions is crucial from a therapeutic perspective. The blue region suggests potential resistance scenarios, while the green and yellow regions offer opportunities for targeted interventions. The boundary representing the minimum population holds significance, indicating conditions conducive to maximizing therapeutic efficacy. The symmetrical patterns observed in the bifurcation diagram provide a framework for predicting the behavior of the system. The interplay between SOX2 expression level and the virus bursting-size parameters reveals a symmetric pattern in the stability region of equilibrium points.

## 4. Discussion

In this study, we established a simple ODE mathematical model to understand the interactions among GSCs, infected GSCs, and ZIKV with a primary focus on the role of SOX2 expression level and the bursting size of ZIKV in dynamic populations. We provided analytic work on the existence and boundness of equilibrium points or solutions. The stability of equilibrium points was performed by local stability analysis. As a result, we showed that the stability of equilibrium points is dependent on the basic reproduction number $R_{0}$, such as if (1) $E_{1}\left(1,0,0\right)$ is stable with $R_{0}<1$, and (2) $E_{2}\left(x_{2},y_{2},v_{2}\right)$ becomes stable with $R_{0}>1$. Sensitivity analysis was evaluated, and the Runge–Kutta 2nd order method was used for numerical simulations.

In our experimental results, we identified two threshold values of SOX2 expression level where SOX2 expression can change from (1) free ZIKV equilibrium point to equilibrium point with ZIKV (transcritical bifurcation) and (2) steady state solutions to oscillations (Hopf bifurcation). We explored how bursting size and SOX2 expression level relate to the efficacy of virotherapy by conducting simulations to analyze stability regions for various equilibrium points on a coordinate system. The effect of SOX2 expression level and bursting size of ZIKV on the stability of equilibrium points is shown in the 2D bifurcation diagram (Figure 5). These two parameters play a crucial role in the dynamics of OVT for therapeutic strategies. The boundary separating green and yellow zones represents the minimum GSC population, offering crucial insights for virotherapy. Transitions between these regions reflect qualitative shifts influenced by SOX2 expression and busting size. Understanding these stability regions is essential for developing effective virotherapy strategies and optimizing therapeutic outcomes.

Mathematical modeling serves as a powerful tool in the development and optimization of therapeutic strategies against many human diseases, including brain tumors [43–45]. Moreover, it has the potential to provide new insights, hypotheses, and experimental directions, ultimately leading to the development of personalized cancer therapies [43–45]. Our study aimed to develop a mathematical model to precisely predict the optimal levels of SOX2 and bursting size for achieving successful virotherapy outcomes against GSCs. The high recurrence and therapy resistance of human glioblastoma may be attributed to the presence of GSCs in GBM, which often accompany high expression levels of the transcription factor SOX2. The Zika viruses are known to target GSCs via SOX2-integrin-mediated infection [27,29]. Therefore, SOX2 can be used as a prediction marker for successful virotherapy as the expression level of SOX2 in GSCs can predict their susceptibility to ZIKV infection and subsequent virotherapy response.

Although our study focused on the aspect of the role of SOX2 expression levels and ZIKV infection, the limitations may include the exclusion of immune responses in our modeling approaches. During oncolytic virotherapy, immune responses, including the activation of natural killer (NK) cells, may influence the anti-tumor efficacy of virotherapy [37]. Our previous study established a mathematical model to assess the fine balance between viral replication rates and NK cell activities during virotherapy. Additionally, recent research has shown that ZIKV E protein co-localizes with SOX2, generating a long-term memory for antitumor immune response [46]. Conducted on mice, this study showed that mice treated with a combination of ZIKV lived significantly longer than other groups, surviving for more than 120 days after treatment [46]. Moreover, ZIKV treatment not only elevated cytotoxic T cell infiltration but also enhanced the antitumor immune response within GSCs by recruiting and activating T cells [46]. In a recent study by Garcia et al., the attenuated Zika viral strain ZOL-1 was utilized in a mouse model of GBM [47]. Their findings underscore the importance of molecular characterization of the target cells for successful virotherapy, as they found two different responsive cancer groups, showing that some patient-derived cancer cells responded better than others [47]. These studies emphasize the necessity for further investigation into the correlation between immune responses and viral infection to achieve maximum efficacy in ZIKV-mediated virotherapy.

## 5. Conclusions

Our mathematical model determined the optimal SOX2 levels for efficient and enhanced virotherapy by identifying critical threshold levels of both SOX2 and viral replication. Given that SOX2 levels in human glioblastoma stem cells are critical for successful ZIKV infection and replication, our model enables precise prediction of the success rates of ZIKV-targeted GSC lysis. This study highlights the potential for developing personalized virotherapies based on a patient’s SOX2 expression levels in their GSCs. Since immune responses may significantly impact the efficacy and outcomes of oncolytic virotherapy, future studies should focus on elucidating the optical conditions for recruiting immune cells, such as cytotoxic T cells, to the infected GSCs and glioblastoma cells to maximize the effects of virotherapy against GBM.

## Figures and Tables

**Table 1.** The model parameters.

Parameter	Description	Value	Units	References
λ	GSC growth rate	0.2971	Million cells/day	[28]
α	Infection rate of ZIKV	0.0504	1/day virus	[28]
$\delta_{1}$	Death rate of infected GSCs	0.3841	1/day	[28]
$\delta_{2}$	Clearance rate of ZIKV	0.125	1/day	[28]

## Data Availability

The MATLAB code will be available upon request.

## References

[1] Czarnywojtek A; Borowska M; Dyrka K; Van Gool S; Sawicka-Gutaj N; Moskal J; Koscinski J; Graczyk P; Halas T; Lewandowska AM; Glioblastoma Multiforme: The Latest Diagnostics and Treatment Techniques. Pharmacology 2023, 108, 423–431, doi:10.1159/000531319. 37459849

[2] Davis ME Glioblastoma: Overview of Disease and Treatment. Clin J Oncol Nurs 2016, 20, S2–8, doi:10.1188/16.CJON.S1.2-8. PMC5123811 27668386

[3] Hanif F; Muzaffar K; Perveen K; Malhi SM; Simjee Sh U Glioblastoma Multiforme: A Review of its Epidemiology and Pathogenesis through Clinical Presentation and Treatment. Asian Pac J Cancer Prev 2017, 18, 3–9, doi:10.22034/APJCP.2017.18.1.3. 28239999 PMC5563115

[4] Paolillo M; Boselli C; Schinelli S Glioblastoma under Siege: An Overview of Current Therapeutic Strategies. Brain Sci 2018, 8, doi:10.3390/brainsci8010015. PMC5789346 29337870

[5] Anjum K; Shagufta BI; Abbas SQ; Patel S; Khan I; Shah SAA; Akhter N; Hassan SSU Current status and future therapeutic perspectives of glioblastoma multiforme (GBM) therapy: A review. Biomed Pharmacother 2017, 92, 681–689, doi:10.1016/j.biopha.2017.05.125. 28582760

[6] Young RM; Jamshidi A; Davis G; Sherman JH Current trends in the surgical management and treatment of adult glioblastoma. Ann Transl Med 2015, 3, 121, doi:10.3978/j.issn.2305-5839.2015.05.10. 26207249 PMC4481356

[7] Biserova K; Jakovlevs A; Uljanovs R; Strumfa I Cancer Stem Cells: Significance in Origin, Pathogenesis and Treatment of Glioblastoma. Cells 2021, 10, doi:10.3390/cells10030621. PMC8000844 33799798

[8] Alves ALV; Gomes INF; Carloni AC; Rosa MN; da Silva LS; Evangelista AF; Reis RM; Silva VAO Role of glioblastoma stem cells in cancer therapeutic resistance: a perspective on antineoplastic agents from natural sources and chemical derivatives. Stem cell research & therapy 2021, 12, 206, doi:10.1186/s13287-021-02231-x. 33762015 PMC7992331

[9] Gimple RC; Bhargava S; Dixit D; Rich JN Glioblastoma stem cells: lessons from the tumor hierarchy in a lethal cancer. Genes Dev 2019, 33, 591–609, doi:10.1101/gad.324301.119. 31160393 PMC6546059

[10] Louis DN; Perry A; Reifenberger G; von Deimling A; Figarella-Branger D; Cavenee WK; Ohgaki H; Wiestler OD; Kleihues P; Ellison DW The 2016 World Health Organization Classification of Tumors of the Central Nervous System: a summary. Acta Neuropathol 2016, 131, 803–820, doi:10.1007/s00401-016-1545-1. 27157931

[11] Henriksson R; Asklund T; Poulsen HS Impact of therapy on quality of life, neurocognitive function and their correlates in glioblastoma multiforme: a review. Journal of neuro-oncology 2011, 104, 639–646, doi:10.1007/s11060-011-0565-x. 21468776 PMC3170120

[12] Sundar SJ; Hsieh JK; Manjila S; Lathia JD; Sloan A The role of cancer stem cells in glioblastoma. Neurosurgical focus 2014, 37, E6, doi:10.3171/2014.9.FOCUS14494. 25434391

[13] Huang Z; Liu M; Huang Y Oncolytic therapy and gene therapy for cancer: recent advances in antitumor effects of Newcastle disease virus. Discov Med 2020, 30, 39–48. 33357361

[14] Kazemi Shariat Panahi H; Dehhaghi M; Lam SS; Peng W; Aghbashlo M; Tabatabaei M; Guillemin GJ Oncolytic viruses as a promising therapeutic strategy against the detrimental health impacts of air pollution: The case of glioblastoma multiforme. Semin Cancer Biol 2022, 86, 1122–1142, doi:10.1016/j.semcancer.2021.05.013. 34004331

[15] Thorne SH; Hwang TH; O’Gorman WE; Bartlett DL; Sei S; Kanji F; Brown C; Werier J; Cho JH; Lee DE; Rational strain selection and engineering creates a broad-spectrum, systemically effective oncolytic poxvirus, JX-963. J Clin Invest 2007, 117, 3350–3358, doi:10.1172/JCI32727. 17965776 PMC2040321

[16] Ferrucci PF; Pala L; Conforti F; Cocorocchio E Talimogene Laherparepvec (T-VEC): An Intralesional Cancer Immunotherapy for Advanced Melanoma. Cancers (Basel) 2021, 13, doi:10.3390/cancers13061383. PMC8003308 33803762

[17] Todo T; Ito H; Ino Y; Ohtsu H; Ota Y; Shibahara J; Tanaka M Intratumoral oncolytic herpes virus G47∆ for residual or recurrent glioblastoma: a phase 2 trial. Nat Med 2022, 28, 1630–1639, doi:10.1038/s41591-022-01897-x. 35864254 PMC9388376

[18] Ling AL; Solomon IH; Landivar AM; Nakashima H; Woods JK; Santos A; Masud N; Fell G; Mo X; Yilmaz AS; Clinical trial links oncolytic immunoactivation to survival in glioblastoma. Nature 2023, 623, 157–166, doi:10.1038/s41586-023-06623-2. 37853118 PMC10620094

[19] de Noronha L; Zanluca C; Burger M; Suzukawa AA; Azevedo M; Rebutini PZ; Novadzki IM; Tanabe LS; Presibella MM; Duarte Dos Santos CN Zika Virus Infection at Different Pregnancy Stages: Anatomopathological Findings, Target Cells and Viral Persistence in Placental Tissues. Front Microbiol 2018, 9, 2266, doi:10.3389/fmicb.2018.02266. 30337910 PMC6180237

[20] Figueiredo CP; Barros-Aragao FGQ; Neris RLS; Frost PS; Soares C; Souza INO; Zeidler JD; Zamberlan DC; de Sousa VL; Souza AS; Zika virus replicates in adult human brain tissue and impairs synapses and memory in mice. Nat Commun 2019, 10, 3890, doi:10.1038/s41467-019-11866-7. 31488835 PMC6728367

[21] Halani S; Tombindo PE; O’Reilly R; Miranda RN; Erdman LK; Whitehead C; Bielecki JM; Ramsay L; Ximenes R; Boyle J; Clinical manifestations and health outcomes associated with Zika virus infections in adults: A systematic review. PLoS Negl Trop Dis 2021, 15, e0009516, doi:10.1371/journal.pntd.0009516. 34252102 PMC8297931

[22] Li H; Hu Y; Huang J; Feng Y; Zhang Z; Zhong K; Chen Y; Wang Z; Huang C; Yang H; Zika virus NS5 protein inhibits cell growth and invasion of glioma. Biochem Biophys Res Commun 2019, 516, 515–520, doi:10.1016/j.bbrc.2019.06.064. 31230744

[23] Li J; Meng Q; Zhou X; Zhao H; Wang K; Niu H; Wang Y Gospel of malignant Glioma: Oncolytic virus therapy. Gene 2022, 818, 146217, doi:10.1016/j.gene.2022.146217. 35093451

[24] Mazar J; Brooks JK; Peloquin M; Rosario R; Sutton E; Longo M; Drehner D; Westmoreland TJ The Oncolytic Activity of Zika Viral Therapy in Human Neuroblastoma In Vivo Models Confers a Major Survival Advantage in a CD24-dependent Manner. Cancer Res Commun 2024, 4, 65–80, doi:10.1158/2767-9764.CRC-23-0221. 38214542 PMC10775766

[25] Mazar J; Li Y; Rosado A; Phelan P; Kedarinath K; Parks GD; Alexander KA; Westmoreland TJ Zika virus as an oncolytic treatment of human neuroblastoma cells requires CD24. PLoS One 2018, 13, e0200358, doi:10.1371/journal.pone.0200358. 30044847 PMC6059425

[26] Francipane MG; Douradinha B; Chinnici CM; Russelli G; Conaldi PG; Iannolo G Zika Virus: A New Therapeutic Candidate for Glioblastoma Treatment. Int J Mol Sci 2021, 22, doi:10.3390/ijms222010996. PMC8537796 34681654

[27] Wang S; Zhang Q; Tiwari SK; Lichinchi G; Yau EH; Hui H; Li W; Furnari F; Rana TM Integrin αvβ5 Internalizes Zika Virus during Neural Stem Cells Infection and Provides a Promising Target for Antiviral Therapy. Cell Rep 2020, 30, 969–983.e964, doi:10.1016/j.celrep.2019.11.020. 31956073 PMC7293422

[28] Zhu Z; Gorman MJ; McKenzie LD; Chai JN; Hubert CG; Prager BC; Fernandez E; Richner JM; Zhang R; Shan C; Zika virus has oncolytic activity against glioblastoma stem cells. J Exp Med 2017, 214, 2843–2857, doi:10.1084/jem.20171093. 28874392 PMC5626408

[29] Zhu Z; Mesci P; Bernatchez JA; Gimple RC; Wang X; Schafer ST; Wettersten HI; Beck S; Clark AE; Wu Q; Zika Virus Targets Glioblastoma Stem Cells through a SOX2-Integrin α(v)β(5) Axis. Cell Stem Cell 2020, 26, 187–204.e110, doi:10.1016/j.stem.2019.11.016. 31956038 PMC9628766

[30] Al-Tuwairqi SM; Al-Johani NO; Simbawa EA Modeling dynamics of cancer virotherapy with immune response. Advances in Difference Equations 2020, 2020, 438, doi:10.1186/s13662-020-02893-6.

[31] Kim PS; Crivelli JJ; Choi IK; Yun CO; Wares JR Quantitative impact of immunomodulation versus oncolysis with cytokine-expressing virus therapeutics. Math Biosci Eng 2015, 12, 841–858, doi:10.3934/mbe.2015.12.841. 25974336

[32] Tian JP The replicability of oncolytic virus: defining conditions in tumor virotherapy. Math Biosci Eng 2011, 8, 841–860, doi:10.3934/mbe.2011.8.841. 21675814

[33] Wodarz D Computational modeling approaches to the dynamics of oncolytic viruses. Wiley Interdiscip Rev Syst Biol Med 2016, 8, 242–252, doi:10.1002/wsbm.1332. 27001049 PMC4896310

[34] Elaiw AM; Al Agha AD Analysis of a delayed and diffusive oncolytic M1 virotherapy model with immune response. Nonlinear Analysis: Real World Applications 2020, 55, 103116, doi:10.1016/j.nonrwa.2020.103116.

[35] Zhao J; Tian JP Spatial Model for Oncolytic Virotherapy with Lytic Cycle Delay. Bull Math Biol 2019, 81, 2396–2427, doi:10.1007/s11538-019-00611-2. 31089864 PMC8890613

[36] Wang Y; Tian JP; Wei J Lytic cycle: A defining process in oncolytic virotherapy. Applied Mathematical Modelling 2013, 37, 5962–5978, doi:10.1016/j.apm.2012.12.004.

[37] Kim D; Shin D-H; Sung CK The Optimal Balance between Oncolytic Viruses and Natural Killer Cells: A Mathematical Approach. Mathematics 2022, 10, 3370.

[38] Kim Y; Yoo JY; Lee TJ; Liu J; Yu J; Caligiuri MA; Kaur B; Friedman A Complex role of NK cells in regulation of oncolytic virus-bortezomib therapy. Proc Natl Acad Sci U S A 2018, 115, 4927–4932, doi:10.1073/pnas.1715295115. 29686060 PMC5948955

[39] Phan TA; Tian JP The Role of the Innate Immune System in Oncolytic Virotherapy. Comput Math Methods Med 2017, 2017, 6587258, doi:10.1155/2017/6587258. 29379572 PMC5742943

[40] Senekal NS; Mahasa KJ; Eladdadi A; de Pillis L; Ouifki R Natural Killer Cells Recruitment in Oncolytic Virotherapy: A Mathematical Model. Bull Math Biol 2021, 83, 75, doi:10.1007/s11538-021-00903-6. 34008149

[41] Alvarado AG; Thiagarajan PS; Mulkearns-Hubert EE; Silver DJ; Hale JS; Alban TJ; Turaga SM; Jarrar A; Reizes O; Longworth MS; Glioblastoma Cancer Stem Cells Evade Innate Immune Suppression of Self-Renewal through Reduced TLR4 Expression. Cell Stem Cell 2017, 20, 450–461.e454, doi:10.1016/j.stem.2016.12.001. 28089910 PMC5822422

[42] Beards CF 5 - Automatic control systems. In Engineering Vibration Analysis with Application to Control Systems, Beards CF, Ed.; Butterworth-Heinemann: London, 1995; pp. 171–279.

[43] Harpold HL; Alvord EC Jr.; Swanson KR The evolution of mathematical modeling of glioma proliferation and invasion. J Neuropathol Exp Neurol 2007, 66, 1–9, doi:10.1097/nen.0b013e31802d9000. 17204931

[44] Jordao G; Tavares JN Mathematical models in cancer therapy. Biosystems 2017, 162, 12–23, doi:10.1016/j.biosystems.2017.08.007. 28866047

[45] Quaranta V; Weaver AM; Cummings PT; Anderson AR Mathematical modeling of cancer: the future of prognosis and treatment. Clin Chim Acta 2005, 357, 173–179, doi:10.1016/j.cccn.2005.03.023. 15907826

[46] Chen L; Zhou C; Chen Q; Shang J; Liu Z; Guo Y; Li C; Wang H; Ye Q; Li X; Oncolytic Zika virus promotes intratumoral T cell infiltration and improves immunotherapy efficacy in glioblastoma. Mol Ther Oncolytics 2022, 24, 522–534, doi:10.1016/j.omto.2022.01.011. 35229030 PMC8851082

[47] Garcia G Jr.; Chakravarty N; Paiola S; Urena E; Gyani P; Tse C; French SW; Danielpour M; Breunig JJ; Nathanson DA; Differential Susceptibility of Ex Vivo Primary Glioblastoma Tumors to Oncolytic Effect of Modified Zika Virus. Cells 2023, 12, doi:10.3390/cells12192384. PMC10572118 37830597

